# Calmodulin is involved in the dual subcellular location of two chloroplast proteins

**DOI:** 10.1074/jbc.RA119.010846

**Published:** 2019-10-02

**Authors:** Lucas Moyet, Daniel Salvi, Imen Bouchnak, Stéphane Miras, Laura Perrot, Daphné Seigneurin-Berny, Marcel Kuntz, Norbert Rolland

**Affiliations:** Laboratoire de Physiologie Cellulaire & Végétale, Université Grenoble Alpes, INRA, CNRS, CEA, IRIG–LPCV, 38000 Grenoble, France

**Keywords:** chloroplast, subcellular fractionation, subcellular organelle, calmodulin (CaM), plant molecular biology, plant biochemistry, Chloroplast envelope, Subcellular location

## Abstract

Cell compartmentalization is an essential process by which eukaryotic cells separate and control biological processes. Although calmodulins are well-known to regulate catalytic properties of their targets, we show here their involvement in the subcellular location of two plant proteins. Both proteins exhibit a dual location, namely in the cytosol in addition to their association to plastids (where they are known to fulfil their role). One of these proteins, ceQORH, a long-chain fatty acid reductase, was analyzed in more detail, and its calmodulin-binding site was identified by specific mutations. Such a mutated form is predominantly targeted to plastids at the expense of its cytosolic location. The second protein, TIC32, was also shown to be dependent on its calmodulin-binding site for retention in the cytosol. Complementary approaches (bimolecular fluorescence complementation and reverse genetics) demonstrated that the calmodulin isoform CAM5 is specifically involved in the retention of ceQORH in the cytosol. This study identifies a new role for calmodulin and sheds new light on the intriguing CaM-binding properties of hundreds of plastid proteins, despite the fact that no CaM or CaM-like proteins were identified in plastids.

## Introduction

In plants, it is largely accepted that import pathways that target proteins into and across the plastid envelope are critical for plant development by regulating the response to physiological and metabolic changes within the cell (1–4). On the other hand, plant cells have developed numerous mechanisms for dual cellular location of proteins, especially plastid proteins. Independent gene duplication events have allowed development of gene families through sequential evolutionary events to target alternative subcellular locations that deviate from the ancestral one (5). Alternatively, a single gene copy can generate alternative mRNA transcripts and protein isoforms. Dual targeting of proteins to chloroplasts and cytosol or mitochondria were shown to be determined by alternative N-terminal presequences, being primarily regulated at the transcriptional level through alternative transcription initiation or transcript splicing, followed by alternative translation initiation (6–8). Furthermore, there is a relatively high frequency (over 100 known cases) of proteins containing ambiguous presequences for dual targeting to mitochondria and chloroplasts (9, 10). Other proteins, containing both an N-terminal chloroplast targeting signal and a nuclear localization signal, are dual targeted to the chloroplast and the nucleus (11). Some studies support a retrograde protein translocation mechanism in which these proteins are first targeted to plastids, processed to the mature form, and then relocated to the nucleus (12). In other words, in the wild context of evolution, plants evolved complementary subsets of strategies for dual targeting of chloroplast proteins.

A plastid protein, termed ceQORH (AT4G13010), originally predicted as a quinone oxido-reductase homolog, was recently shown to reduce long-chain, stress-related oxidized lipids that are spontaneously produced in the chloroplast envelope from the unstable allene oxide formed in the biochemical pathway leading to 12-oxo-phytodienoic acid, a precursor of the defense hormone jasmonate (13, 14). Targeted studies (15) and proteomic data (16–18) unambiguously localized ceQORH in the inner membrane of the chloroplast envelope. Consistent with these observations, this protein was imported into purified intact chloroplasts using *in vitro* import assays (19–21). However, while analyzing its *in vivo* subcellular location in leaf cells, we formerly observed that this protein was not exclusively targeted to plastids (19) but was also partly localized at the periphery of plant cells and in some locally concentrated dots in the cytosol. Thus, in cells from *Arabidopsis* leaves, ceQORH shows a complex subcellular location pattern: in the plastid envelope (following import into plastids) and outside plastids (implying that plastid import did not occur). Here, we show that the calmodulin isoform CaM5^7^ is a key player for this dual location, thus providing a so-far-unanticipated role for the intriguing CaM-binding properties of hundreds of plastid proteins (22), despite the fact that no CaM or CaM-like proteins were identified in plastids.

## Results

### The plant ceQORH protein interacts with calmodulin

**Figure 1.**
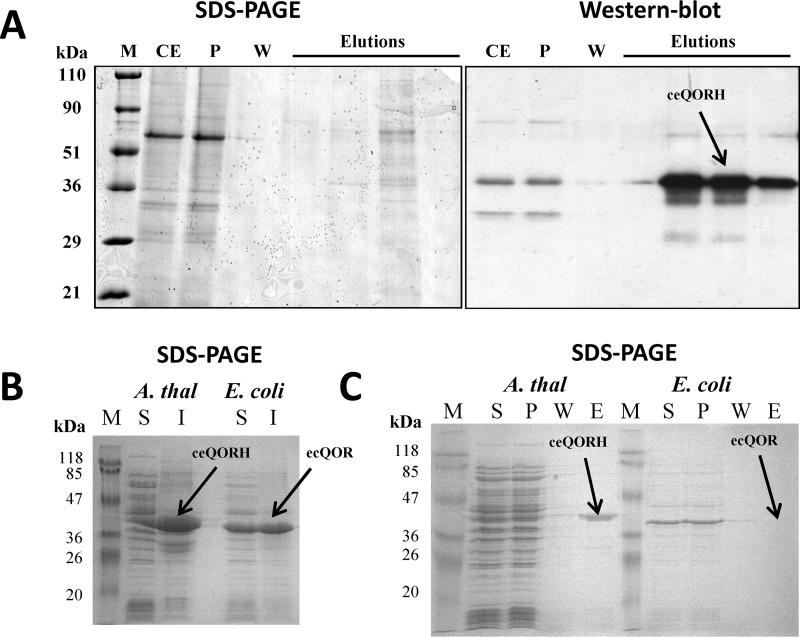
**Interaction of the natural plant ceQORH protein with calmodulin.** *A*, affinity purification of *Arabidopsis* ceQORH from crude plant cell extracts. Purification was performed on a CaM affinity resin (Stratagene). *Lane M*, prestained protein molecular weight markers; *lane CE*, crude solubilized plant proteins diluted in CaM-binding buffer containing 0.1% Nonidet P-40; *lane P*; unbound proteins; *lane W*, wash; *lane E*, four successive elution fractions are presented. *B*, production of recombinant *Arabidopsis* ceQORH and *E. coli* K12 QOR (ecQOR) proteins in *E. coli* SDS-PAGE analysis of crude bacterial extracts containing *Arabidopsis* ceQORH or *E. coli* ecQOR proteins. *S*, soluble fraction of the crude bacterial proteins; *I*, insoluble fraction of the crude bacterial proteins (inclusion bodies). *C*, affinity purification of *Arabidopsis* ceQORH and *E. coli* ecQOR produced in bacteria (see *B*). Purification was performed on a CaM affinity resin (Stratagene). As a control, the bacterial ecQOR protein was also tested. *Lane S*, soluble bacterial proteins diluted in CaM-binding buffer; *lane P*, unbound proteins; *lane W*, wash; *lane E*, pooled elution fractions. Note that the recombinant *Arabidopsis* ceQORH protein interacts with the CaM affinity resin (and is thus eluted from the column), whereas this is not the case for the recombinant *E. coli* ecQOR protein.

In the present study, we provide several lines of evidence demonstrating the specific CaM-binding property of ceQORH. First, the natural plant ceQORH was enriched in the fraction eluted from a calmodulin-affinity resin when compared with its level in crude cell extracts (Fig. 1*A*). Then we compared the CaM-binding properties of the recombinant ceQORH protein produced in *Escherichia coli* with those of the endogenous protein ecQOR (as a negative control), the closest bacterial homolog of plant ceQORH (Fig. 1*B*). As demonstrated in Fig. 1*C*, although the plant ceQORH is detected in the elution fraction of CaM affinity chromatography, its bacterial homolog is only detected in the pass-through fraction, indicating that it does not bind CaM.

### The CaM-binding domain is located in the C terminus of ceQORH

**Figure 2.**
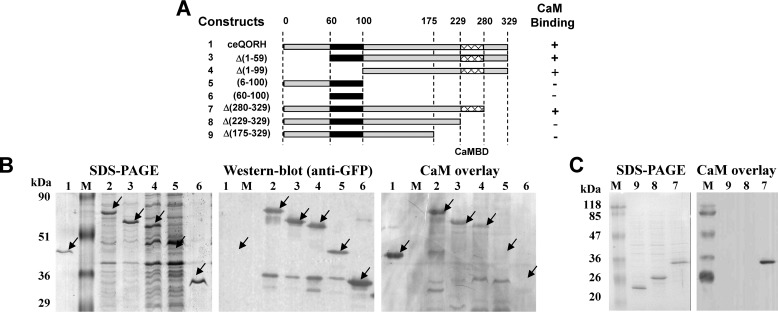
**The CaM-binding domain is located in the C terminus of ceQORH.** *A*, scheme of the successive deletions and constructs used to localize the CaM-binding domain in the ceQORH sequence. Sequences of the various deletions in ceQORH are indicated. *Construct 1*, ceQORH protein; *construct 2*, ceQORH-GFP (full-length ceQORH protein fused to GFP); *construct 3*, D(1–59)ceQORH-GFP (ceQORH lacking 60 residues in N terminus fused to GFP); *construct 4*, D(1–99)ceQORH-GFP (ceQORH lacking 100 residues in N terminus fused to GFP); *construct 5*, (6–100)ceQORH-GFP (ceQORH lacking both 6 residues in N terminus and 229 residues in C terminus fused to GFP); *construct 6*, (60–100)ceQORH-GFP (ceQORH lacking both 60 residues in N terminus and 229 residues in C terminus fused to GFP); *construct 7*, D(280–329)ceQORH-GFP (ceQORH lacking 49 residues in N terminus fused to GFP); *construct 8*, D(229–329)ceQORH-GFP (ceQORH lacking 100 residues in N terminus fused to GFP); and *construct 9*, D(175–329)ceQORH-GFP (ceQORH lacking 146 residues in N terminus fused to GFP). The CAM-binding domain in the ceQORH sequence is deduced from ability of the various constructs to interact with CaM (+ means CaM-binding, and − means lack of CaM binding) and supported by results of experiments shown in *B* and *C. B*, SDS-PAGE. *Lanes 1–6* are crude bacterial extracts containing recombinant ceQORH fusions as described for *A. Lanes M*, prestained protein molecular weight markers. Western blotting was performed using a primary antibody raised against GFP (1/70,000), an anti-rabbit HRP-conjugated secondary antibody (1/10,000) and ECL. For CaM overlay, after transfer, washing and saturation, the membrane was incubated in the hybridization solution containing 0.1 μg/ml biotinylated CAM. Detection of the bound CaM protein was performed using a streptavidin–HRP conjugate and ECL. *C*, successive deletions in the C terminus of ceQORH to localize its CaM-binding domain. For SDS-PAGE, *lanes 7–9* are crude bacterial extracts containing recombinant ceQORH fusions as described in *A. M*, prestained protein molecular weight markers. With CaM overlay, after transfer, washing, and saturation, the membrane was incubated in the hybridization solution containing 0.3 μg/ml of CaM-HRP conjugate. Detection of the bound CaM protein was performed using ECL.

**Figure 3.**
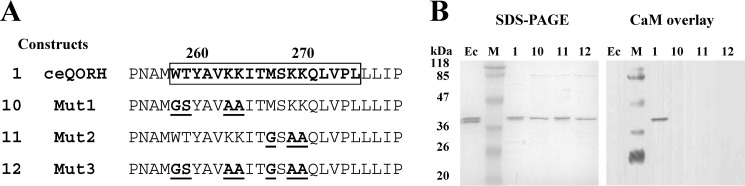
**Targeted mutagenesis identifies essential residues required for CaM-binding properties of ceQORH.** *A*, scheme of the residues selected for targeted mutagenesis of the ceQORH sequence. The CAM-binding region in the ceQORH sequence is deduced from successive deletions of ceQORH domains (see Fig. 2). *B*, SDS-PAGE and CaM overlay. *Lanes Ec*, purified recombinant QOR from *E. coli* K12 used as a negative control; *lanes M*, prestained protein molecular weight markers; *lanes 1*, purified recombinant WT form of ceQORH from *A. thaliana* used as a positive control; *lanes 10*, purified mutagenized form of *Arabidopsis* ceQORH mutant 1; *lanes 11*, purified mutagenized form of *Arabidopsis* ceQORH mutant 2; *lanes 12*, purified mutagenized form of *Arabidopsis* ceQORH mutant 3. Note that, in Mut2, mutagenesis of only three residues is sufficient to abolish interaction of ceQORH with CaM.

To establish the location of the CaM-binding domain of ceQORH, we first performed successive deletions of the ceQORH protein (Fig. 2*A*) and expressed these constructs in *E. coli* (Fig. 2*B*). Using these experiments, we were able to demonstrate that this CaM-binding domain is located in the C terminus of ceQORH (Fig. 2, *B* and *C*) between residues 229 and 280 of the protein. In this region, we searched for a putative CaM-binding site within this domain using the CaM prediction program (23), which identified (see Fig. S1) the following candidate motif: ^255^AMWTYAVKKITMSKKQLVPLLL^277^. CaM-binding peptides have the potential to fold into a basic, amphiphilic α-helix (see Ref. 24) in which hydrophobic and charged residues are predicted to be essential for formation of this helix. We thus decided to modify some residues by site-directed mutagenesis (Fig. 3*A* and Fig. S2) within the helix or adjacent to this helix according to the recently established 3D structure of ceQORH (14). Three of these mutants could be isolated in which mutagenesis of positively charged and hydrophobic residues abolishes the interaction of ceQORH with CaM (Fig. 3*B*). As deduced from CaM overlay experiments performed on purified recombinant proteins (Fig. 3*B*), mutagenesis of only three residues abolishes the CaM-binding properties of ceQORH. This mutated form, termed Mut2-ceQORH, was thus considered as the best tool for further approaches.

### CaM binding is neither essential for ceQORH targeting to chloroplasts nor required for the specific location of ceQORH to the plastid envelope

**Figure 4.**
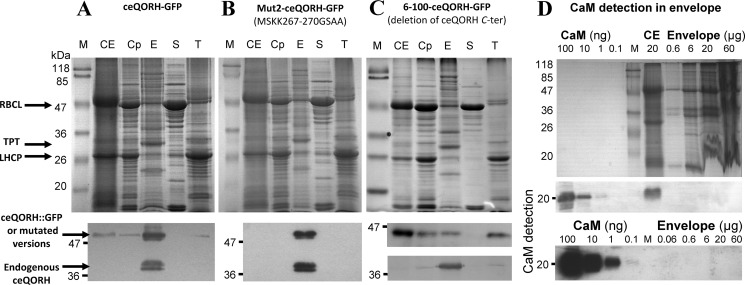
**The ceQORH CaM-binding domain is neither essential for ceQORH targeting to parenchymal cell chloroplasts nor required for specific localization of ceQORH to the plastid envelope.** *A–C*, lack of CaM-binding domain does not affect envelope localization of ceQORH. Cell fractionation was performed on plants stably expressing in *A*, ceQORH-GFP (ceQORH fused to GFP); in *B*, Mut2-ceQORH-GFP (ceQORH mutant affected in CaM-binding properties fused to GFP); and in *C*, 6–100-ceQORH-GFP (ceQORH lacking its 197 C-terminal residues, including the CaM-binding domain, fused to GFP). *Lanes M*, prestained protein molecular weight markers; *lanes CE*, crude cell extract; *lanes Cp*, chloroplast extract; *lanes E*, envelope; *lanes S*, stroma; *lanes T*, thylakoids. Note that to limit artifacts resulting from overexpression of the various ceQORH constructs, transgenic plants were selected for expression levels of recombinant proteins similar to endogenous ceQORH level. Each lane contains 20 μg of proteins. *RBCL*, large subunit of RuBisCO (stroma marker). *LHCP*, light-harvesting complex proteins (thylakoid membrane marker). *TPT*, Triose-P/phosphate translocator (envelope marker). Western blotting analyses were performed using the antibody raised against ceQORH. *D*, purified chloroplast envelope fractions do not contain detectable levels of CaM. *CaM*, 100 to 0.1 ng of purified recombinant CaM1 from *Arabidopsis. Lane M*, prestained protein molecular weight markers. *CE*, crude cell extract, envelope (0.6–60 μg of envelope proteins). Note that a second CaM detections experiment is shown, using longer exposure time to improve sensitivity of ECL detection.

To determine whether the CaM-binding properties of ceQORH are responsible for the targeting of this protein to the chloroplast envelope, we established stable *Arabidopsis* transformants expressing one truncated form (lacking its C terminus, *i.e.* construct 5 in Fig. 2*A*) and one mutated form (Mut2-ceQORH lacking CaM-binding properties, *i.e.* construct 11 in Fig. 3*A*) of ceQORH and examined the subplastidial location of these mutated forms. Note that to limit artifacts resulting from overexpression of the various ceQORH constructs, transgenic plants were selected for expression levels of recombinant proteins similar to endogenous ceQORH level (see Fig. 4, *A* and *B*, *lower panels*). Strikingly, whereas the full-length ceQORH is enriched in purified chloroplast envelope fractions (Fig. 4*A*), ceQORH lacking its C terminus also accumulates in thylakoid membranes at a similar level (Fig. 4*C*). The simplest interpretation is that the C terminus of ceQORH (which contains its CaM-binding domain) also promotes selective location at the envelope membrane. However, Mut2-ceQORH only lacking CaM-binding properties, is also targeted to the chloroplast and only detected in the purified envelope fraction and not in thylakoids (Fig. 4*B*), similarly to the endogenous ceQORH protein. This clearly demonstrates that the CaM-binding domain of ceQORH is not essential, *in planta*, for its targeting to the chloroplast or for its specific association to the chloroplast envelope.

To examine the possibility that ceQORH could nevertheless bind a calmodulin within the chloroplast envelope, we then assessed whether CaM isoforms were present in purified envelope fractions. In good agreement with previous MS-based analyses targeting purified chloroplast envelope fractions (17, 18), we could not detect signals associated with CaM isoforms at the envelope level (Fig. 4*D*). Furthermore, a function of CaM associated to ceQORH independently of the envelope membrane was further provided through quantification of CaM within purified envelope fractions. Indeed, through dilution and Western blotting analyses, we observed that although representing ∼1/2000 (∼10 ng CaM in 20 μg of crude leaf extract) of all leaf proteins (Fig. 4*D*, *bottom panel*), CaM should represent less than 1/600,000 (less than 0.1 ng of CaM in 60 μg of envelope proteins) of all envelope proteins. Knowing that ceQORH represents 2.5% of envelope proteins (17), a ceQORH/CaM ratio above 2000 would thus be poorly compatible with a meaningful role for CaM binding to ceQORH at the chloroplast envelope level.

### A high-molecular-mass CaM isoform is enriched in membrane fractions of epidermal cells compared with crude leaf extract

To further elucidate the role of the CAM-binding properties of ceQORH, it was necessary to identify which CaM isoform interacts with ceQORH *in planta*. Indeed, plant cells contain seven classical (short, *i.e.* 16 kDa) CaM isoforms and tens of CaM-like proteins (25, 26).

**Figure 5.**
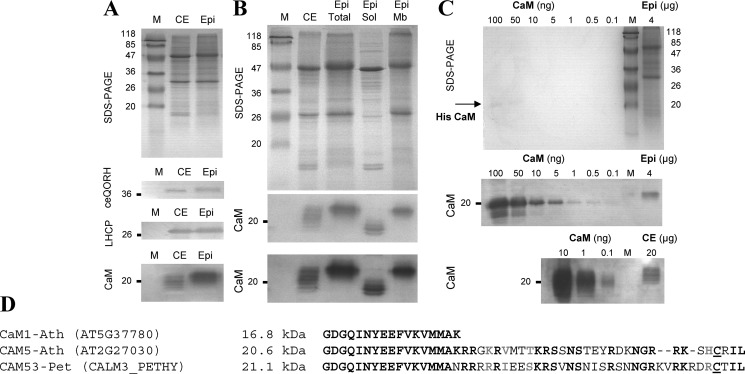
**Membranes fractions of epidermal cells from *Arabidopsis* leaves are enriched in a high-molecular-weight CaM isoform when compared with crude leaf extract.** *A*, detection of CaM isoforms in crude leaf extracts (*CE*) and epidermal tissue from *Arabidopsis* leaves. Western blotting was performed using antibodies raised against ceQORH, LHCP, and CaM-767. *B*, molecular mass of the CaM isoform enriched in epidermal cells is above (∼20 kDa) the one expected for classical (short) CaM isoforms (∼16 kDa). *Epi*, 5 μg of proteins from crude epidermal extract from *Arabidopsis* leaves. Fractionation of membrane and soluble fractions of epidermal tissue reveals that the high-molecular-weight (>20 kDa) CaM isoform is bound to membranes. *M*, prestained molecular mass markers; *CE*, 15 μg of proteins from crude leaf extracts from *Arabidopsis*; epidermal tissue (*Epi*) 15 μg of proteins from crude epidermal extract from *Arabidopsis* leaves; *Sol*, 15 μg of soluble proteins from epidermal tissue; *Mb*, 15 μg of membrane proteins from epidermal tissue. Note that a second CaM detection experiment is shown using longer exposure time to improve sensitivity of ECL detection. *C*, CaM is more abundant in epidermal cells (1/400 to 1/800 of all epidermal proteins) when compared with crude leaf extract (1/2000 to 1/20,000 of all cell proteins). *CaM*, 100 to 0.1 ng of purified recombinant CaM1 from *Arabidopsis. M*, prestained protein molecular weight markers; *Epi*, 4 μg of proteins from crude epidermal extract; *CE*, 20 μg of proteins from crude leaf extract. *D*, alignment of C termini from classical short CaM isoform (*e.g.* CaM1) with the CaM isoform identified in the PM from petunia (CaM53-Pet) and its closest homolog in *Arabidopsis* (CaM5-Ath). Note that CaM5 from *Arabidopsis* and CaM53 from petunia contain an additional C terminus sequence when compared with short CaM isoforms (CaM1). Conserved residues are *bold* (*black* for identity and *gray* for similarity). The *underlined* C residue is the CaM53 isoprelynation site.

Having previously noted that ceQORH was mainly present at the periphery of plant cells in epidermal tissues (19), we decided to assess the abundance of CaM isoforms in epidermal tissue compared with crude leaf extracts. As seen in Fig. 5*A*, the CaM signal was enriched in epidermal tissue compared with whole leaf extract. Strikingly, this signal in epidermal cells was of higher molecular mass (*i.e.* >20 kDa) than classical (shorter) CaM isoforms (*i.e.* 16 kDa). This high-molecular-mass signal was also enriched in the membrane fraction of epidermal cells (Fig. 5*B*), whereas the soluble fraction contained CaM isoforms of lower molecular mass. Finally, this high-molecular-mass CaM signal was 10–20 times more enriched in epidermal cells (∼5 ng CaM in 4 μg of crude epidermal extract, *i.e.* 1/1000 of total epidermal proteins) compared with crude leaf extract (∼1 ng of CaM in 20 μg of crude leaf extract, *i.e.* 1/20,000 of total leaf proteins) (Fig. 5*C*). In screening databases for a membrane-bound CaM-like isoform of higher molecular mass than classical (short) CaM isoforms, we identified CaM53 from petunia (CALM3_PETHY), a CaM isoform that contains an additional C-terminal sequence compared with short CaM isoforms (*e.g.* CaM1, AT5G37780) (Fig. 5*D*). A close homolog of this CaM53 protein from petunia is present in *Arabidopsis*, and this isoform is named CaM5 (AT2G27030.3). Compared with other CaM isoforms, this CaM5 isoform contains an additional C terminus sequence that is highly conserved (Fig. 5*D*) with CaM53 from petunia (CALM3_PETHY). Notably, using AT2G27030 as a query in the Uniprot database, only a classical (short) version (149 amino acids, molecular mass of 16,820 Da) of CaM5 is referenced (Q682T9 or TCH1 or CALM5_ARATH). However, in the TAIR database, a query using the term AT2G27030 results in the gene model AT2G27030.3, which encodes a 181-amino acid protein with a molecular mass of 20,575.9 Da (*i.e.* similar to CaM53 from petunia).

### CaM5 and ceQORH interact in planta

**Figure 6.**
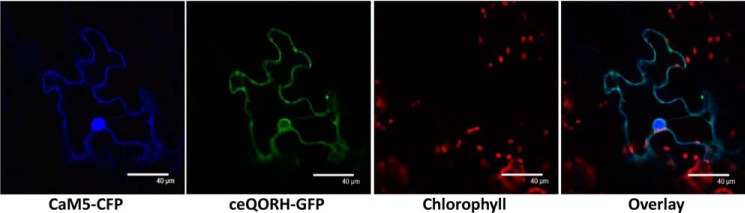
**CaM5 and ceQORH colocalize in epidermal cells.** Confocal microscopy was performed on plants stably expressing ceQORH-GFP (ceQORH fused to GFP) and transiently expressing CaM5 fused to cyan fluorescent protein (*CaM5-CFP*). *Chlorophyll*, chlorophyll autofluorescence. *Overlay*, overlay of all three channels. *Bar*, 40 μm. Note that although CaM5 and ceQORH are colocated at the periphery of leaf cells, only CaM5 accumulates within the nucleus.

**Figure 7.**
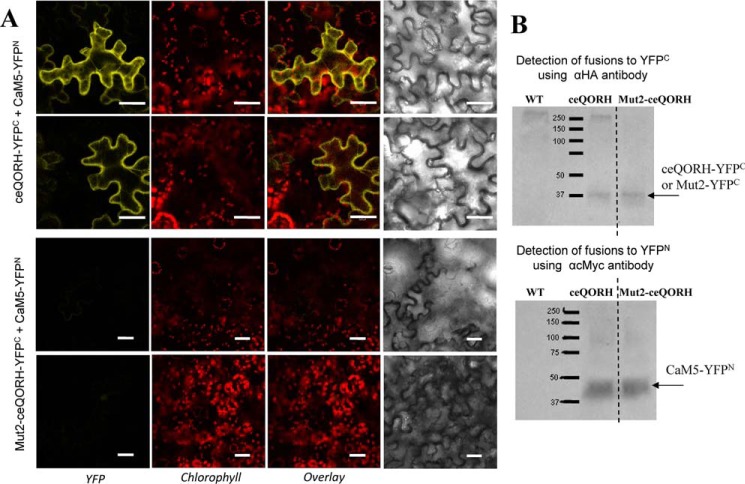
**Identification of the CaM isoform interacting with ceQORH: CaM5 and ceQORH interact in epidermal cells.** *A*, confocal imaging (BiFC) performed on plants transiently expressing either ceQORH–YFPC (ceQORH fused to the C terminus of YFP) or Mut2–ceQORH–YFPC (Mut2 version of ceQORH fused to the C terminus of YFP) and CaM5-YFPN (CaM5 sequence fused to the N terminus of YFP). *YFP*, fluorescence of YFP resulting from BiFC. *Chlorophyll*, chlorophyll autofluorescence. *Overlay*, overlay of the two channels. *Bar*, 40 μm. *B*, Western blotting analyses performed to validate expression of YFP fusions. Crude cell extracts from WT plants were included as negative controls. Note that although both ceQORH–YFPC and Mut2–ceQORH–YFPC accumulate at a similar level in plant cells, BiFC is only detected using the WT ceQORH version (*i.e.* containing its CaM-binding domain). Note that BiFC is mostly detected at the periphery of leaf cells, *i.e.* where CaM5 and ceQORH colocalize within epidermal cells (see Fig. 6).

Having identified CaM5 (AT2G27030.3) as a candidate for the most abundant (and high-molecular-mass CaM) isoform detected in epidermal cells (*i.e.* where ceQORH is mostly located at the periphery of the cells), we were encouraged to pursue its analysis using confocal imaging. First, ceQORH and CaM5 were colocalized at the periphery of plant cells in epidermal cells (Fig. 6). One can also note that CaM5 also accumulates within the nucleus as previously observed for its close ortholog CaM53 from petunia (27). Note that ceQORH is only present at the periphery of the nucleus but does not accumulate within the nucleus. To establish whether ceQORH and CaM5 interact *in planta*, we then used bimolecular fluorescence complementation (BiFC) (Fig. 7*A*). Because of its lack of CaM-binding properties, the Mut2-ceQORH mutant was used as a negative control during these experiments (Fig. 7*A*, *lower panel*). Strikingly, although both ceQORH and Mut2-ceQORH accumulate at similar levels in plant cells (Fig. 7*B*), BiFC was only detected using the WT version of ceQORH (*i.e.* containing the CaM-binding domain) and mostly at the periphery of leaf cells (*i.e.* where CaM5 and ceQORH were colocalized in Fig. 6).

### ceQORH lacking its CaM-binding domain is targeted to chloroplasts

**Figure 8.**
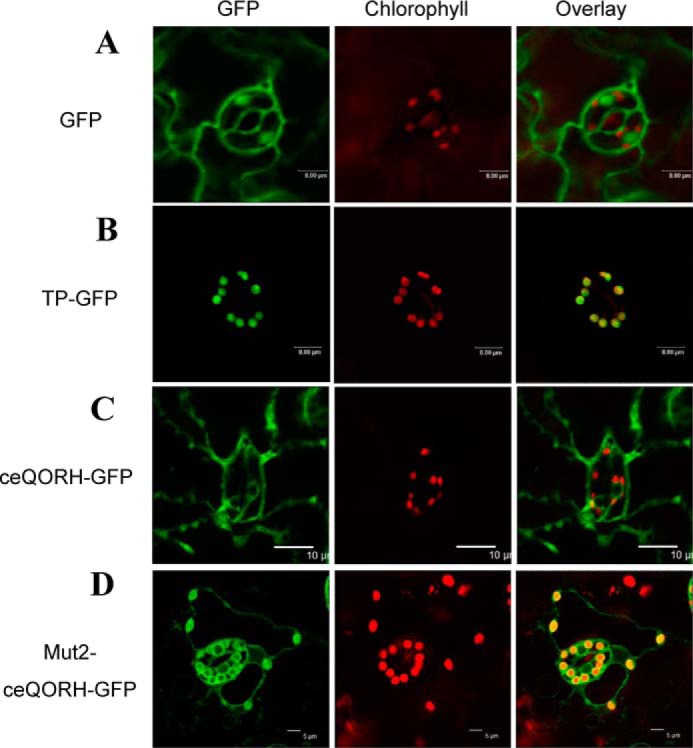
**ceQORH lacking its CaM-binding domain is targeted to chloroplasts in epidermal cells.** Confocal microscopy was performed on plants stably expressing the following: *A*, GFP (GFP alone as a negative control of plastid localization; *bar*, 8 μm). *B*, TP-GFP (transit peptide of the small subunit of RuBisCO fused to GFP as a positive control of plastid localization; *bar*, 8 μm). *C*, ceQORH-GFP (plant ceQORH fused to GFP; *bar*, 10 μm). *D*, Mut2-ceQORH-GFP (ceQORH lacking its CaM-binding domain; *bar*, 5 μm). *GFP*, GFP fluorescence. *Chlorophyll*, chlorophyll autofluorescence. *Overlay*, overlay of all three channels.

We then expressed Mut2-ceQORH (*i.e.* ceQORH lacking its CaM-binding domain) *in planta* and analyzed its subcellular localization compared with that of the WT form of ceQORH. Again, to limit artifacts resulting from overexpression of the various GFP fusions, the selected transgenic plants were the above-used ones (Fig. 4, *A* and *B*, *lower panels*), in which the expression levels of GFP fusions are similar to endogenous ceQORH level. Interestingly, whereas ceQORH-GFP is never detected in plastids in epidermal cells (Fig. 8*C*; see also Fig. S3 for additional images), Mut2-ceQORH accumulates at the periphery of plastids (Fig. 8*D*), thus most likely in their envelope membrane, in agreement with Fig. 4*B*. The simplest interpretation is that, *in planta*, the CaM-binding domain of ceQORH promotes an alternative subcellular location of ceQORH via its retention in the cytosol by CaM5.

### CaM5 is the primary regulator of ceQORH retention outside plastids

**Figure 9.**
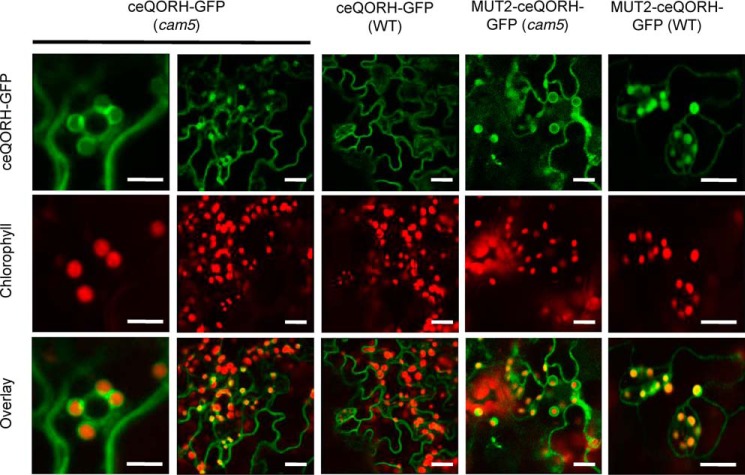
**CaM5 is the CaM isoform that controls retention of ceQORH at the periphery of plant cells: ceQORH is targeted to the chloroplast envelope in epidermal cells of the *cam5* mutant.** Confocal microscopy was performed on both WT plants and *cam5* mutant stably expressing ceQORH-GFP (plant ceQORH fused to GFP) or Mut2-ceQORH-GFP. *ceQORH-GFP*, GFP fluorescence. *Chlorophyll*, chlorophyll autofluorescence. *Overlay*, overlay of the two channels. The *bar* indicates 5 μm in the *left column* and 10 μm in the *four right columns*.

As stated above, plant cells contain seven classical (short) CaM isoforms and tens of CaM-like proteins (25, 26). It was thus required to definitively establish that CaM5 plays an essential role in controlling the subcellular location of ceQORH and to exclude the involvement of other CaM isoforms in this process. We used a CaM5 T-DNA insertion mutant, termed *cam5-4* by Al-Quraan *et al.* (28), which was shown to be a true KO mutant (lacking CaM5 mRNA). Subcellular localization of ceQORH (and Mut2-ceQORH as a control) in this loss-of-function *cam5* mutant demonstrated that ceQORH was now targeted to the chloroplast in epidermal cells (Fig. 9; see also Fig. S4), in contrast to the location pattern (exclusively at the periphery of the cells) observed above in control WT cells. In agreement with its lost CaM-binding properties, Mut2-ceQORH was also detected in plastids in both WT plant and *cam5* mutant (Fig. 9 and Fig. S5). This result confirms that CaM5 is indeed a crucial actor of the observed alternative location of ceQORH. However, other actors may also contribute to this regulatory process as suggested by the observation that a certain level of ceQORH is still observed at the cell periphery for ceQORH in the *cam5* mutant and Mut2-ceQORH in both WT and *cam5* backgrounds (Figs. S3–S5).

### CaM control of subcellular location is not limited to ceQORH

**Figure 10.**
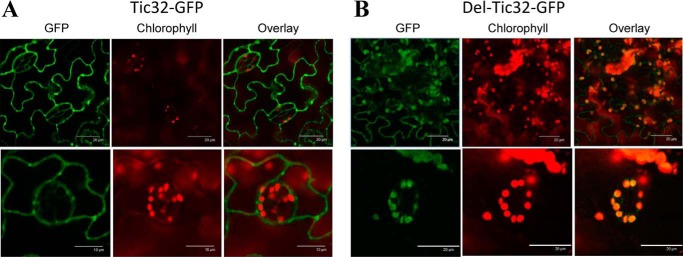
**CaM5 control of chloroplast targeting is not limited to ceQORH.** *A*, like ceQORH, Tic32 accumulates at the periphery of epidermal cells when containing its CaM-binding domain. *B*, Tic32 lacking its CaM-binding domain is targeted to chloroplasts in epidermal cells. Confocal microscopy was performed on plants stably expressing Tic32-GFP (Tic32 fused to GFP; *bar*, 10 or 20 μm) and Del-Tic32-GFP (Tic32 protein lacking its C terminus; *bar*, 20 μm). *GFP*, GFP fluorescence. *Chlorophyll*, chlorophyll autofluorescence. *Overlay*, overlay of the two channels.

To examine whether this calmodulin-dependent mechanism plays a more global role, we used Tic32, an inner envelope membrane protein, whose CaM-binding capacity was previously demonstrated and whose CaM-binding domain was previously localized in its C terminus (29). More recently, this Tic32 protein was also retrieved in the elution of a CaM-affinity resin starting from solubilized chloroplast envelope proteins, and its CaM-binding capacity was confirmed during the same work using overlay assays (22). Interestingly, when expressed *in planta*, Tic32 was also detected at the periphery of epidermal cells and in a few dotted structures distinct from plastids (Fig. 10*A*), as observed for ceQORH (Figs. 6–9). Similar to Mut2-ceQORH, Tic32 lacking its C terminus (*i.e.* its CaM-binding domain) was mostly detected in plastids (Fig. 10*B*). Thus, these results indicate that, like ceQORH, the alternative subcellular location of the chloroplast envelope protein Tic32 is also CaM-dependent.

## Discussion

During evolution, plants evolved complementary subsets of strategies for dual targeting of proteins. This work documents a novel strategy allowing a dual plastid–cytosol location of plant proteins. This evidence is achieved despite the fact that ceQORH has been shown to be imported into plastids in *in vitro* import experiments using isolated chloroplasts. We propose here that, when tightly bound to CaM5, ceQORH cannot reach plastids. The direct role of a calmodulin-like protein in controlling ceQORH location is shown by the fact that the loss of the CaM-binding properties of ceQORH (*i.e.* Mut2-ceQORH) greatly abolishes retention outside of the plastids and instead leads to a plastid location of this protein. The expression of ceQORH in the *cam5* mutant proves that CaM5, a calmodulin-like protein, is a crucial actor involved in this phenomenon.

It should be mentioned here that CAM5 binding to ceQORH does not prevent its import into the chloroplast through interaction with a canonical chloroplast transit peptide because ceQORH is devoid of such an N-terminal extension and is known to be imported via a “noncanonical” pathway (19). The latter pathway is poorly understood but must involve an internal domain of the ceQORH to be imported into the chloroplast (19).

**Figure 11.**
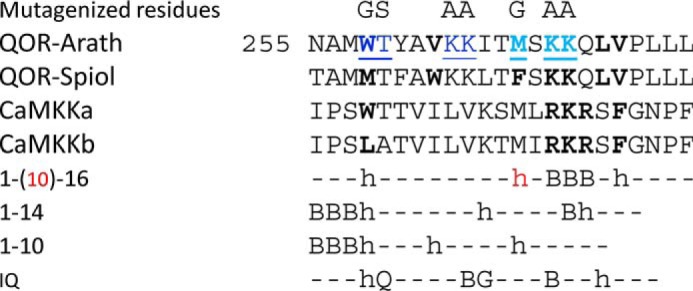
**Comparison of the CaM-binding domain of ceQORH from *Arabidopsis* and spinach to the major classes of CaM-binding domains.** *h* indicates a hydrophobic residue, and *B* indicates a basic residue. In the classical nomenclature of these classes, each number is positioning a hydrophobic residue in their consensus sequences (see the work of Yap *et al.* (23) and Tidow and Nissen (30)). The ceQORH peptides show closest similarity to the 1-16 class, which contains two isoforms of a calcium/calmodulin dependent protein kinase kinase (CaMKKa and CaMKKb). Other classes are the 1-14 class (comprising the 1-14, 1-8-14, basic 1-8-14, and 1-5-8-14 subclasses), the 1-10 class (comprising the 1-10, 1-5-10, basic 1-5-10, and hydrophilic 1-4-10 subclasses), and the IQ class, to which the ceQORH CaM-binding domain appears more distally related. Residues that were mutagenized in ceQORH are indicated (*dark blue* in Mut1-ceQORH, *light blue* in Mut2-ceQORH, and both *dark* and *light blue* in Mut3-ceQORH).

Interestingly, the bacterial homolog of ceQORH does not appear to bind calmodulin (Figs. 1 and 3), despite the fact that both proteins are highly conserved and share a similar charge and size, as well as other biophysical properties (14, 15). Moreover, we previously showed that this bacterial ecQOR protein is not targeted to chloroplasts *in planta* (15). Thus, these observations show that, during the evolution of this protein from bacteria to eukaryotic cells, ceQORH has acquired both an internal plastid targeting domain and a CaM-binding domain (Fig. S1). The latter shows the closest similarity to the 1-16 class of CaM-binding domains (23, 30) which contains two isoforms of a calcium/calmodulin–dependent protein kinase kinase (Fig. 11). This CaM-binding motif is located within an α-helix, which appears to be accessible in the 3D structure (14) of the ceQORH from *Arabidopsis thaliana* (Fig. S2). The positions of the residues, whose mutagenesis abolished CaM-binding capacity of ceQORH, demonstrate that not only this amphiphilic α-helix structure but also adjacent basic residues are essential for this interaction.

Another condition for CaM5 to trap ceQORH outside plastids is that it must be sufficiently abundant. This is the case in epidermal cells (Fig. 5). Epidermal cells are actually a convenient system to identify this phenomenon because ceQORH is mainly trapped outside the plastids because CaM5 is abundant, whereas ceQORH is predominantly localized in plastids in its absence (*i.e. cam5* mutant) or when the CaM-binding site of ceQORH is lost (*i.e.* Mut2-ceQORH mutant). However, this phenomenon also exists in mesophyll cells where, however, the balance between plastid and nonplastid locations is in favor of the former (and therefore relocation is not that easy to monitor).

We also showed that this mechanism also influence the subcellular location of another “noncanonical” plastid protein, namely Tic32, which also lacks a cleavable chloroplast transit peptide (31). However, it is important to note here that it cannot be concluded that all plastid proteins lacking a typical transit peptide will follow the same rule. Indeed, although recent observations (21) strongly support that ceQORH does not engage with the trimeric TOC159/75/34 complex for translocation into the chloroplasts, a result that is consistent with previous work (19), this recent work also demonstrated that, en route to the plastid, FSD1, which lacks a cleavable cTP, recognizes a specific receptor (Toc132 and Toc120) and engages with components of the general import pathway, as do the majority of plastid preproteins (21). In other words, although large-scale proteomic studies identified hundreds of plastid proteins lacking a predicted cTP (16–18, 32, 33), the use of such alternative trafficking pathways to the plastid might not be a general rule in all cases where a cTP is missing.

It is well-known that CaM proteins have no catalytic activity but rather act as sensor relays that regulate the catalytic properties of downstream targets (27). Our results demonstrates, for the first time, that a specific CaM isoform is also involved in the subcellular location of its target(s). Understanding of the global role of this mechanism will require further specific and large-scale analyses. Indeed, more than 200 CaM-binding proteins were recently identified from different *Arabidopsis* and spinach chloroplast subfractions (22). Most of them had never been shown to interact with CaM before. The role of their CaM-binding property thus remains to be elucidated. These CaM targets are involved in all main plastid functions, such as photosynthesis (*e.g.* PSAN, STN8), amino acid synthesis metabolism (*e.g.* ArgJ, TS2, DAHPS3, PRT2), lipid and hormone metabolism (*e.g.* Lox2, ceQORH), protein targeting and folding (*e.g.* SecA, Tic32), DNA transcription (*e.g.* PTAC17), thylakoid biogenesis (*e.g.* ViPP1), etc. Furthermore, some of these proteins showed CaM-binding properties that are either dependent or independent of the presence of calcium (22), suggesting another level of regulation through various controlled interactions.

Finally, many other questions emerge from this work. For example, the role of CaM5 might not be limited to proteins targeted to plastids and may encompass dual targeting of proteins in other cell compartments. Furthermore, whether such a subcellular control of protein location involving CaM isoforms exists in other eukaryotic kingdoms also remains to be investigated.

## Experimental procedures

### Plant material and standard growth conditions

*Arabidopsis* plants, Wassilevskija background (WS), were germinated in Petri dishes containing solidified medium (Murashige and Skoog, 1% (w/v) sucrose, and 1% (w/v) agarose) for 2 weeks before being transferred to soil. Plants were then grown in growth rooms at 23 °C (12-h light cycle) with a light intensity of 100 μmol·m^−2^·s^−1^.

### Accession numbers

The sequence data from this article can be found in the Arabidopsis Genome Initiative or GenBank^TM^/EMBL databases under the following accession numbers: ceQORH (At4g13010), CaM5 (AT2G27030.3), and Tic32 (At4g23430).

### Genotyping of the cam5 mutant from Arabidopsis

The *Arabidopsis* homozygous mutant (SALK_027181) affected in the cam5 gene (AT2G27030.3) is a T-DNA insertion mutant termed cam5-4 (28), which shows no visible phenotype. The presence of the T-DNA (and its homozygous state) was verified using the following flanking primers: CaM5fwd (CTCGGCAGCTGAGTTAAGACATG) and CaM5rev (GCTGCCATAACTCTCTTCCCTC) coupled to T-DNA primers LB (GGCAATCAGCTGTTGCCCGTCTCACTGGTG) and RB (GCTCATGATCAGATTGTCGTTTCCCGCCTT).

### Purification of chloroplasts and chloroplast subfractions from Arabidopsis

All operations were carried out at 0–5 °C. Percoll-purified chloroplasts were obtained from 200–300 g of *A. thaliana* leaves. Chloroplast fractions were purified from these purified chloroplasts as previously described (34) and stored in liquid nitrogen until use. The protein content of fractions was estimated using the Bio-Rad protein assay reagent (35).

### Purification of crude cell extracts and epidermal tissue from Arabidopsis leaves

Whole-cell proteins from *Arabidopsis* leaves were extracted from 2–4-week-old leaves using a small plastic potter and an Eppendorf tube in 150 μl of extraction buffer containing 30 mm tetrasodium pyrophosphate, 100 mm Tris-HCl (pH 6.8), and 1% (w/v) SDS. The crude extract was centrifuged for 5 min at 13,200 rpm and 4 °C. Because of the presence of a high concentration of detergent, the supernatant resulting from this centrifugation step contains whole-cell proteins (including highly hydrophobic membrane proteins). Purification of epidermal tissues from *Arabidopsis* leaves was performed by peeling the epidermis of *Arabidopsis* leaves using a thin forceps used for plant crosses. Crude epidermal extracts were obtained using the procedure described above and the same extraction buffer. Membrane and soluble fractions of crude cell extracts were obtained as previously described (19).

### Production of recombinant His-CaM1 and of rabbit polyclonal antibodies directed against Arabidopsis CaM1 protein

The CaM1 isoform (AT5G37780) was selected because of its high sequence conservation with other CaM isoforms (CaM1 is 100% identical to CaM4; 97% identical to CaM2, CaM3, CaM6, CaM7, and the first 149 residues of CaM5; and 76% identical to CaM8 and CaM-Like11). Cloning of the *Arabidopsis* cDNA coding for the CAM1 protein was performed by RT-PCR using pFU ultra high-fidelity DNA polymerase (Stratagene) starting from a homemade *Arabidopsis* cDNA library. The primer GAAGAACATATGGCGGATCAACTC was designed to introduce an NdeI restriction site (underlined residues) at the 5′ end of the DNA fragment coding for CAM1. The primer ATCACCTGGATCCAATCACTTAGC was designed to introduce a BamHI restriction site (underlined residues) at the 3′ end of the DNA fragment coding for CaM1. The amplified fragments were cloned in pBluescriptSK^−^. The insert was then digested with NdeI and BamHI and inserted into the expression vector pET-15b (Novagen). The resulting expression plasmids allowed the expression of N-terminal His-tagged versions of the CaM1 protein. The purified protein was desalted (PD-10 column, Pharmacia) and stored at −80 °C. The purified recombinant His-CaM1 was used as a control (from 100 to 0.1 ng) to estimate the content of the natural CaM proteins in crude leaf extracts, epidermal fractions, or purified chloroplast envelope membranes. Two independent rabbit polyclonal antibodies (including CaM-767) raised against the *Arabidopsis* recombinant CaM1 protein were obtained from the Elevage Scientifique des Dombes (catalog no. F-01400; Châtillon sur Chalaronne, France).

### SDS-PAGE and Western blotting analyses

SDS-PAGE analyses were performed as described by Chua (36) using prestained protein molecular weight markers (Fermentas). For Western blotting analyses, gels were transferred to a nitrocellulose membrane (catalog no. BA85; Schleicher & Schuell). ceQORH was detected using a previously described ceQORH rabbit polyclonal antiserum raised against the recombinant *Arabidopsis* ceQORH produced in *E. coli* (15) at a 1/5000 dilution. The antibody raised against LHCP was provided by Dr. Olivier Vallon (Institut de Biologie Physico-Chimique Paris) and used at a 1/20,000 dilution. The secondary antibody for ceQORH and LHCP was an anti-rabbit HRP-conjugated secondary antibody (Interchim) used at a 1/10,000 dilution. GFP was detected using a primary antibody raised against GFP (Euromedex) at a 1/70,000 dilution and an anti-mouse HRP-conjugated secondary antibody (Interchim). Detection of the proteins was performed using ECL. CaM transfer and detection require specific conditions. First, the transfer requires 80 min (at 80 V) in a specific buffer (20 mm Tris-HCl, 15 mm glycine, 2 mm CaCl_2_, 20% (v/v) ethanol). The membrane was then washed for 5 min in TBST and incubated for 1 h in TBST containing 5% (w/v) defatted milk. Incubation with the primary antibody at a 1/1500 dilution requires an overnight incubation at 4 °C. The membrane was then washed three times for 10 min in TBST and incubated for 1 h in TBST containing the secondary antibody (anti-rabbit fused to HRP; Interchim) at a 1/10,000 dilution. The membrane was then washed three times for 10 min in TBST before the proteins were detected using ECL. Notably, because of the 97% sequence identity between the two isoforms, the rabbit polyclonal antibody CaM-767 raised against the *Arabidopsis* CaM1 isoform cross-reacts with all *Arabidopsis* CaM isoforms, including CaM5.

### Production of vectors for expression of the full-length and truncated forms of ceQORH in E. coli to identify its CaM-binding domain

Several constructs used to express ceQORH and several truncated forms in *E. coli* were previously described (15, 19): construct 1, ceQORH; construct 2, ceQORH-GFP (full-length ceQORH protein fused to GFP); construct 3, Δ(1–59)ceQORH-GFP (ceQORH lacking 60 residues in the N terminus fused to GFP); construct 4, Δ(1–99)ceQORH-GFP (ceQORH lacking 100 residues in the N terminus fused to GFP); construct 5, (6–100)ceQORH-GFP (ceQORH lacking both 6 residues in the N terminus and 229 residues in C terminus fused to GFP); and construct 6 (60–100)ceQORH-GFP (ceQORH lacking both 60 residues in the N terminus and 229 residues in the C terminus fused to GFP). Additional constructs were produced, as follows. Construct 7, Δ(280–329)ceQORH-GFP (ceQORH lacking 49 residues in the N terminus fused to GFP), was amplified using the following primers: ceQORH1–175 (TATGCAGTCCAGCTGTAAGAGCGTTGGATC) and ceQORH 1–175 reverse (GATCCAACGCTCTTACAGCTGGACTGCATA). Construct 8, Δ(229–329)ceQORH-GFP (ceQORH lacking 100 residues in the N terminus fused to GFP), was amplified using the following primers: ceQORH 1–229 (GTGGTCCATTGTGCATAAGAGCGTTGGATC) and ceQORH1–229 reverse (GATCCAACGCTCTTATGCACAATGGACCAC). Construct 9, Δ(175–329)ceQORH-GFP (ceQORH lacking 146 residues in the N terminus fused to GFP), was amplified using the following primers: ceQORH1–280 (CTCTTGTTGATCCCAAAATAAGAGCGTTGGATC) and ceQORH1–280 reverse (GATCCAACGCTCTTATTTTGGGATCAACAAGAG). For targeted mutagenesis and the creation of construct 10 (Mut1), construct 11 (Mut2), and construct 12 (Mut3), vectors were created from targeted mutagenesis of available vectors using the QuikChange® site-directed mutagenesis kit from Stratagene. Mut1-ceQORH was obtained using the following primers: mut1qorBamHI (GGGCCTAATGCAATGGGATCCTATGCGGTTGCTGCAATAACCATGTCAAAG) and mut1qorBamHIreverse (CTTTGACATGGTTATTGCAGCAACCGCATAGGATCCCATTGCATTAGGCCC). Mut2-ceQORH was obtained using the following primers: mut2qorBamHI (GTTAAGAAAATAACCGGATCCGCAGCTCAGTTAGTGCCACTC) and mut2qorBamHIreverse (GAGTGGCACTAACTGAGCTGCGGATCCGGTTATTTTCTTAAC). Mut3-ceQORH was obtained using the following primers: mut3qorBamHI (GGGCCTAATGCAATGGGATCCTATGCGGTTGCTGCAATAACCGGGTCAGCAGCTCAGTTAGTGCCACTC) and mut3qorBamHIreverse (GAGTGGCACTAACTGAGCTGCTGACCCGGTTATTGCAGCAACCGCATAGGATCCCATTGCATTAGGCCC).

### Production of recombinant full-length and truncated forms of ceQORH in E. coli

Several constructs used to express full-length ceQORH and truncated forms in *E. coli* were previously described (15, 19). The additional Δ(175–329), Δ(229–329), or Δ(280–329) truncated forms and the constructs corresponding to mutants 1–3 (targeted mutagenesis) were obtained during this work. Briefly, PCR-amplified fragments were digested with NdeI and BamHI and inserted into the expression vector pET-15b (Novagen). For all constructs, after the induction of protein expression for 3 h using isopropyl β-d-thiogalactopyranoside, bacterial pellets (equivalent to 1 ml of cell culture) were solubilized in 100 μl of 10 mm Tris-HCl, pH 6.8. Because GFP was present in all protein fusions, it was possible to estimate the relative expression levels (using Western blotting and GFP antibody detection) obtained for all recombinant proteins. Variation in expression levels was thus controlled before CaM overlay experiments. As a negative control, the bacterial ecQOR protein (from *E. coli* K12) was also produced and tested.

### Affinity purification of recombinant CaM-binding proteins from Arabidopsis or E. coli

Affinity purification of *Arabidopsis* ceQORH from crude cell extracts was performed on crude solubilized plant proteins diluted in CaM-binding buffer (10 mm Tris-HCl, pH 8.0, 150 mm NaCl, 1 mm magnesium acetate, 1 mm imidazole, 2 mm CaCl_2_) containing 0.1% (w/v) Nonidet P-40. CaM-binding proteins were purified using CaM affinity resin (Stratagene) and the small-scale quick batch method according to the manufacturer's instructions with minor modifications. Purification from bacteria started with bacterial pellets (equivalent to 100 ml of cell culture) that were solubilized in 5 ml of CaM-binding buffer. After three rounds (for 2 min) of sonication, cell extracts were centrifuged for 20 min at 5500 rpm at 4 °C. Then Nonidet P-40 (0.1% (w/v) final concentration) was added to the resulting supernatant (5 ml). CaM affinity resin (Stratagene; 40 μl, binding capacity equivalent to 60–100 μg of proteins) was then added to the solution before incubation (stirring wheel) for 1.5 h at 4 °C. The resin was then washed with 5 ml of CaM-binding buffer, and bound proteins were eluted in 250 μl of elution buffer (10 mm Tris-HCl, pH 8.0, 150 mm NaCl, 1 mm magnesium acetate, 1 mm imidazole, 2 mm EGTA, 0.1% (w/v) Nonidet P-40). Purified recombinant proteins were then quantified using the Bio-Rad protein assay reagent (35) before SDS-PAGE analyses and CaM overlay.

### CaM overlay

After SDS-PAGE and transfer, the resulting membrane was first dried and further incubated for 45 min in TBS buffer containing 0.1% (w/v) Triton X-100. The membrane was then incubated for 1 h in TBS buffer containing 0.1% (w/v) Triton X-100 and 5% (w/v) defatted milk followed by 1 h in saturation buffer containing 3% (w/v) gelatin, 50 mm Tris-HCl, pH 7.5, 50 mm MgCl_2_, 150 mm NaCl, and 5 mm CaCl_2_. Hybridization was then performed for 2 h in hybridization buffer containing 1% (w/v) gelatin, 50 mm Tris-HCl, pH 7.5, 50 mm MgCl_2_, 150 mm NaCl, 5 mm CaCl_2_, and 0.1% (w/v) Tween 20) + 0.1 μg/ml biotinylated CaM (Sigma). Detection of the bound CaM protein was then performed using a streptavidin–HRP conjugate (Sigma) and ECL using the manufacturer's instructions. Alternatively, after transfer, washing, and saturation, the membrane was incubated in the hybridization solution containing 0.3 μg/ml of a homemade CaM–HRP conjugate (the recombinant *Arabidopsis* CaM1 (AT5G37780) was covalently coupled to HRP). Detection of the bound CaM protein was then performed using ECL according to the manufacturer's instructions.

### Arabidopsis transformation

WT and transgenic *Arabidopsis* plants (ecotype WS) were transformed by dipping the floral buds of 4-week-old plants into an *Agrobacterium tumefaciens* (C58 strain) solution containing a surfactant (Silwett L-77), according to Clough and Bent (37). Primary transformants were selected on Murashige and Skoog medium containing 100 mg/liter kanamycin. Resistant lines expressing the recombinant protein (as controlled with Western blots using GFP antibody) were selected for further analyses. Primary transformants were then self-pollinated to obtain plants homozygous for insertion.

### Construction of vectors for stable expression of GFP fusions in Arabidopsis

To construct vectors for the expression in *Arabidopsis*, all cDNAs were PCR-amplified using pFU ultra high-fidelity DNA polymerase (Stratagene) starting from a homemade *Arabidopsis* cDNA library. Correct orientation and sequence of the inserted fragment were controlled. Plasmids used for *A. tumefaciens* transformation were prepared using the QIAfilter plasmid midi kit (Qiagen).

#### 

##### ceQORH

The coding region of Arabidopsis ceQORH (AT4G13010) was PCR-amplified using the two flanking primers ceQORHBamHI–N-ter (CCTGGATCCATGGCTGGAAAACTCATG) and ceQORHSacI–C-ter (ACAGAGCTCTTATGGCTCGACAATGATCTTC), and the PCR product was cloned into the pBluescript SK^−^ vector (Stratagene) to create the ceQORH-pKS vector. The BamHI–SacI fragment cleaved from this plasmid was inserted into the BamHI–SacI–digested pEL103 binary vector (kanamycin resistance to transform WT plants), resulting in a vector allowing stable expression of ceQORH-GFP.

##### Mut2-ceQORH

Mut2-ceQORH was produced using targeted mutagenesis of pre-existing constructs using the following primers: mut2qorBamHI (GTTAAGAAAATAACCGGATCCGCAGCTCAGTTAGTGCCACTC) and mut2qorBamHIrevers (GAGTGGCACTAACTGAGCTGCGGATCCGGTTATTTTCTTAAC).

##### TIC32

The coding region of *Arabidopsis* Tic32 (At4g23430) cDNA was PCR-amplified using the two flanking primers Tic32SalI–N-ter (GCTAGTCGACATATGTGGTTTTTTGGATC) and Tic32NcoI–C-ter (TCCTCCATGGAACTGCTTTCTCCTGATTG). This fragment was cloned into the pKS vector (Stratagene) and then transferred to pUC-GFP (see Ref. 15) using the SalI (5′) and NcoI (3′) restriction sites. Then the EcoRI/HindIII cassette was transferred to pEL103, resulting in a vector allowing stable expression of Tic32-GFP.

##### Del-Tic32

Tic32 lacking its C terminus (*i.e.* lacking 27 residues in the C terminus, including the CaM-binding domain; see Ref. 26) was constructed as for Tic32-GFP, except that the primer used to amplify the Tic32 cDNA in 3′ was Del-Tic32NcoI–C-ter (GTATCCATGGCAAGTGGTAATGGTTTAGC).

### Construction of vectors for BiFC

Construction of the plasmids for the expression of ceQORH, Mut2-ceQORH and CaM5 proteins fused to the YFP C terminus or N terminus was performed as follows. The coding regions of candidate proteins were PCR-amplified using two flanking primers, XbaI–N-ter (TCATCTAGAATGGCTGGAAAACTC for ceQORH and Mut2-ceQORH or TCATCTAGAATGGCAGATCAGCTC for CaM5) and SalI–C-ter (CCAGTCGACTGGCTCGACAATGATC for ceQORH and Mut2-ceQORH or CCAGTCGACGAGAATACGGCAGTG for CaM5). The PCR products were cloned into the pBlueScript SK^−^ vector. XbaI–SalI fragments cleaved from this plasmid were inserted into the XbaI–SalI–digested YFP C-terminal or YFP N-terminal reporter plasmid pUC-SPYCE and pUC-SPYNE (38) to create the 35Ω candidate YFP C-terminal/N-terminal vectors. From these constructs, the EcoRI–HindIII fragments were extracted and inserted into the EcoRI–HindIII–digested pEL103 binary vector (kanamycin resistance as a selection marker for transformed plants). The correct orientation and sequence of the inserted fragments were controlled. Constructs were transferred to *A. tumefaciens* strain C58 and used to transform tobacco leaves using agroinfiltration. Plasmids used for *A. tumefaciens* transformation were prepared using the NucleoSpin plasmid kit (Macherey–Nagel). Localization of the GFP and GFP fusions in tobacco leaves was analyzed by confocal fluorescence microscopy 3–4 days after agroinfiltration.

### Confocal microscopy

Fluorescence microscopy was performed with a confocal laser-scanning microscope (TCS-SP2; Leica, Deerfield, IL).

## Author contributions

L. M., D. S., I. B., S. M., L. P., M. K., and N. R. conceptualization; L. M., D. S., I. B., and N. R. resources; L. M., D. S., I. B., D. S.-B., M. K., and N. R. data curation; L. M., D. S.-B., M. K., and N. R. formal analysis; L. M., D. S., I. B., and N. R. supervision; L. M., M. K., and N. R. funding acquisition; L. M., I. B., S. M., M. K., and N. R. validation; L. M., D. S., I. B., S. M., L. P., D. S.-B., M. K., and N. R. investigation; L. M., D. S., I. B., S. M., and N. R. visualization; L. M., D. S., I. B., S. M., L. P., D. S.-B., M. K., and N. R. methodology; L. M., D. S., I. B., M. K., and N. R. writing-original draft; L. M., M. K., and N. R. writing-review and editing.

## Supplementary Material

Supporting Information

## Footnotes

7 The abbreviations used are:

CaM: calmodulin
BiFC: bimolecular fluorescence complementation
LHCP: light-harvesting complex protein
HRP: horseradish peroxidase
YFP: yellow fluorescent protein.
