# Supplementary material for: Calmodulin is involved in the dual subcellular location of two chloroplast proteins
Source: J Biol Chem. 2019 Oct 2;294(46):17543–54. doi: 10.1074/jbc.RA119.010846 (PMC6873194; doi:10.1074/jbc.RA119.010846)
Supplement: Supporting Information [file supp_294_46_17543__index.html]

Calmodulin is involved in the dual subcellular location of two chloroplast proteins — Calmodulin control of cellular location — Calmodulin is involved in the dual subcellular location of two chloroplast proteins — Calmodulin control of cellular location — Supporting Information 

# Calmodulin is involved in the dual subcellular location of two chloroplast proteins

## Supporting Information

- Supporting Information (to be published online) - Supporting Fig 1-5 Supporting information (to be published online)
