# Supplementary material for: Calmodulin is involved in the dual subcellular location of two chloroplast proteins
Source: J Biol Chem. 2019 Oct 2;294(46):17543–54. doi: 10.1074/jbc.RA119.010846 (PMC6873194; doi:10.1074/jbc.RA119.010846)
Supplement: Supporting Information [file supp_RA119.010846_155510_0_supp_404637_pyrd97.pdf]

```
                       10        20        30        40        50        60        70        80
                        |         |         |         |         |         |         |         |
QORH_Arath     ---MAGKLMHALQYNSYGGGAAGLEHVQVPVPTPKSNEVCLKLEATSLNPVDWKIQKGMIRPFLPRKFPCIPATDVAGEV
QORH_Spiol     ---MAAKLMHAIQYSGYGGGTDALKHVEVAVPDPKSDELLLKIEAATLNPIDWKIQKGVLRPLLPRKFPTIPGTDVAGEV
QOR_Ecoli      -------MATRIEFHKHGG-PEVLQAVEFTPADPAENEIQVENKAIGINFIDTYIRSGL---YPPPSLPSGLGTEAAGIV
QOR_YEAST      MKCTIPEQQKVILIDEIGG-YDVIKYEDYPVPSISEEELLIKNKYTGVNYIESYFRKGI---YPCEK-PYVLGREASGTV
Consensus                 i     GG  dvlk v# pvpdp e#Ell knkatg N !# yirkG    ypp k P  lgt#aaG V
Prim.cons.     MKCMA3KLMHAIQY44YGGG4DVLKHVEVPVPDPK2NELLLKN2ATGLNPIDW2I2KG42RPY2PRKFP4I2GT22AGEV

                       90       100       110       120       130       140       150       160
                        |         |         |         |         |         |         |         |
QORH_Arath     VEVGSGVKNFKAGDKVVAVLSHLGGGGLAEFAVATEKLTVKRPQEVGAAEA---AALPVAGLTALQALTNPAGLKLDGTG
QORH_Spiol     VQAGSAVNRFKTGDKVVAVLSHATGGALAEYAVAKENLTVARPPEVSAAEG---AALPVAALTAHQALTQFANIKLDGSG
QOR_Ecoli      SKVGSGVKHIKAGDRVV-Y-AQSALGAYSSVHNIIADKAAILPAAISFEQA---AASFLKGLTVYYLLRKTYEIKPD---
QOR_YEAST      VAKGKGVTNFEVGDQVA-YISNSTFAQYSKISS--QGPVMKLPKGTSDEELKLYAAGLLQVLTALSFTNEAYHVKKG---
Consensus      v vGsgVknfkaGD Vv y s st gays           klP   s e#a   AA  l gLTal  l   y iK d
Prim.cons.     V4VGSGVKNFKAGDKVVA2LSHSTGGA22E4AVA3E4LTVK2P4EVSA2EAKLYAALP2AGLTALQALT4424IKLDG2G

                      170       180       190       200       210       220       230       240
                        |         |         |         |         |         |         |         |
QORH_Arath     KKANILVTAASGGVGHYAVQLAKLANAHVTATCG-ARNIEFVKSLGADEVLDYKTPE--GAALKSPSGKKYDAVVHCANG
QORH_Spiol     ERKNILITAASGGVGHYAVQLAKLGNTHVTATCG-ARNLDFVKGLGADEVLDYKTPE--GASLTSPSGKKYDYVVHGASG
QOR_Ecoli      --EQFLFHAAAGGVGLIACQWAKALGAKLIGTVGTAQKAQSALKAGAWQVINYREEDLVERLKEITGGKKVRVVYDSVGR
QOR_YEAST      --DYVLLFAAAGGVGLILNQLLKMKGAHTIAVASTDEKLKIAKEYGAEYLINASKEDILRQVLKFTNGKGVDASFDSVGK
Consensus           L  AAaGGVGlia QlaK  gah iat gta kl  ak  GA  vi#y  e#     lk t GKkvdav dsvg
Prim.cons.     224NIL4TAA2GGVG22AVQLAKL42AHV2ATCGTAR2L4F2K4LGADEV22YKT2222GA4LKS2SGKK2DAVV2S2GG

                      250       260       270       280       290       300       310       320
                        |         |         |         |         |         |         |         |
QORH_Arath     IPFSVFEPNLSENGKVIDITPGPNAMWTYAVK------KITMSKKQLVPLLLIPK--AENLEFMVNLVKEG--KVKTVID
QORH_Spiol     IPWSTFEPNLSEAGKVIDLTPGPTAMMTFAWK------KLTFSKKQLVPLLLIPK--IPNFEYVVNLVKEK--KLKTVID
QOR_Ecoli      DTWERSLDCLQRRGLMVSFGNSSGAVTGVNLGILNQKGSLYVTRPSLQGYITTREELTEASNELFSLIASGVIKVDVAEQ
QOR_YEAST      DTFEISLAALKRKGVFVSFGNASGLIPPFSITRLSPK-NITLVRPQLYGYIADPEEWKYYSDEFFGLVNSK--KLNIKIY
Consensus      dtfe sl  L r G  !sfgn sga   f    l  k  it  rpqL gyi  pee  e s#e f L! sg  Kl   i
Prim.cons.     2222422PNLS24GKV22F22G2GAM4TFA4K2L22KGK2T4S22QLV222LIP2E24ENSEE42NLVK22VIK2KTVID

                      330       340       350
                        |         |         |
QORH_Arath     SKHPLSKAEDAWAKSIDGHATGKIIVEP--
QORH_Spiol     SKHPLSKGEDAWSRIMGGHATGKIIIEP--
QOR_Ecoli      QKYPLKDAQRAHEILESRATQGSSLL-IP-
QOR_YEAST      KTYPLRDYRTAAADIESRKTVGKLVLEIPQ
Consensus       kyPL da  A a iesr t Gk  leip
Prim.cons.     SK2PLS2AEDAWA4IES2H2TGKIILE2PQ
```

Supplementary Figure 1. Alignment of primary sequences from ceQORH from arabidopsis (AT4G13010 or QORH_ARATH) and spinach (QORH_SPIOL) with their closest homologues from *E. coli* (QOR1_ECOLI) and Yeast (QOR_YEAST). The 60-100 domain of ceQORH is (red box) is essential for its targeting to the chloroplast. All blue boxes (including the bold domain (256-277) in QORH_ARATH) were predicted as putative calmodulin-binding sites using the Calmodulin Target Database (Yap *et al*., 2000).

Yap, K.L., Kim, J., Truong, K. Sherman, M., Yuan, T. Ikura, M. (2000) Calmodulin Target Database. *J Struct Func Genom* (2000) 1: 8-14.

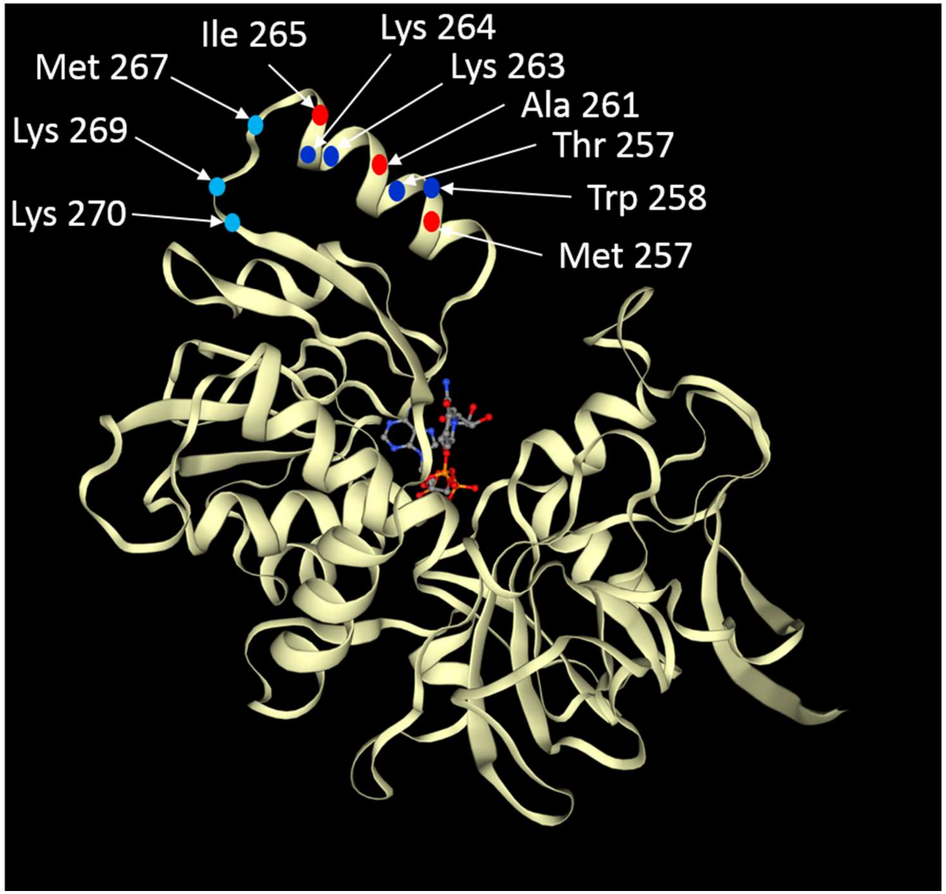

Supplementary Figure 2. Location of the CaM binding motif within the crystal structure (Mas-Y-Mas *et al*., 2017) **of the ceQORH from *Arabidopsis thaliana* bound to NADP**. Note that the CaM binding peptide (256-AMWTYAVKKITMSKKQLVPLLL-277), predicted using the CaM-binding prediction program (http://calcium.uhnres.utoronto.ca/ctdb/ctdb/home.html), folds into a basic, amphiphilic alpha-helix in which hydrophobic and charged residues are predicted to be essential for formation of this helix (see Vetter and Leclerc, 2003). Residues modified by site directed mutagenesis (see Fig. 3A) are indicated in dark blue (for Mut1-ceQORH) and light blue (for Mut2-ceQORH).

Mas Y Mas, S., Curien, G., Giustini, C., Rolland, N., Ferrer, J.L., Cobessi, D. (2017) Crystal Structure of the Chloroplastic Oxoene Reductase ceQORH from *Arabidopsis thaliana*. *Front. Plant Sci*. 8: 329-329

Vetter, S. W. and Leclerc, E. (2003) Novel aspects of calmodulin target recognition and activation. *Eur. J. Biochem.* 270:404-414.

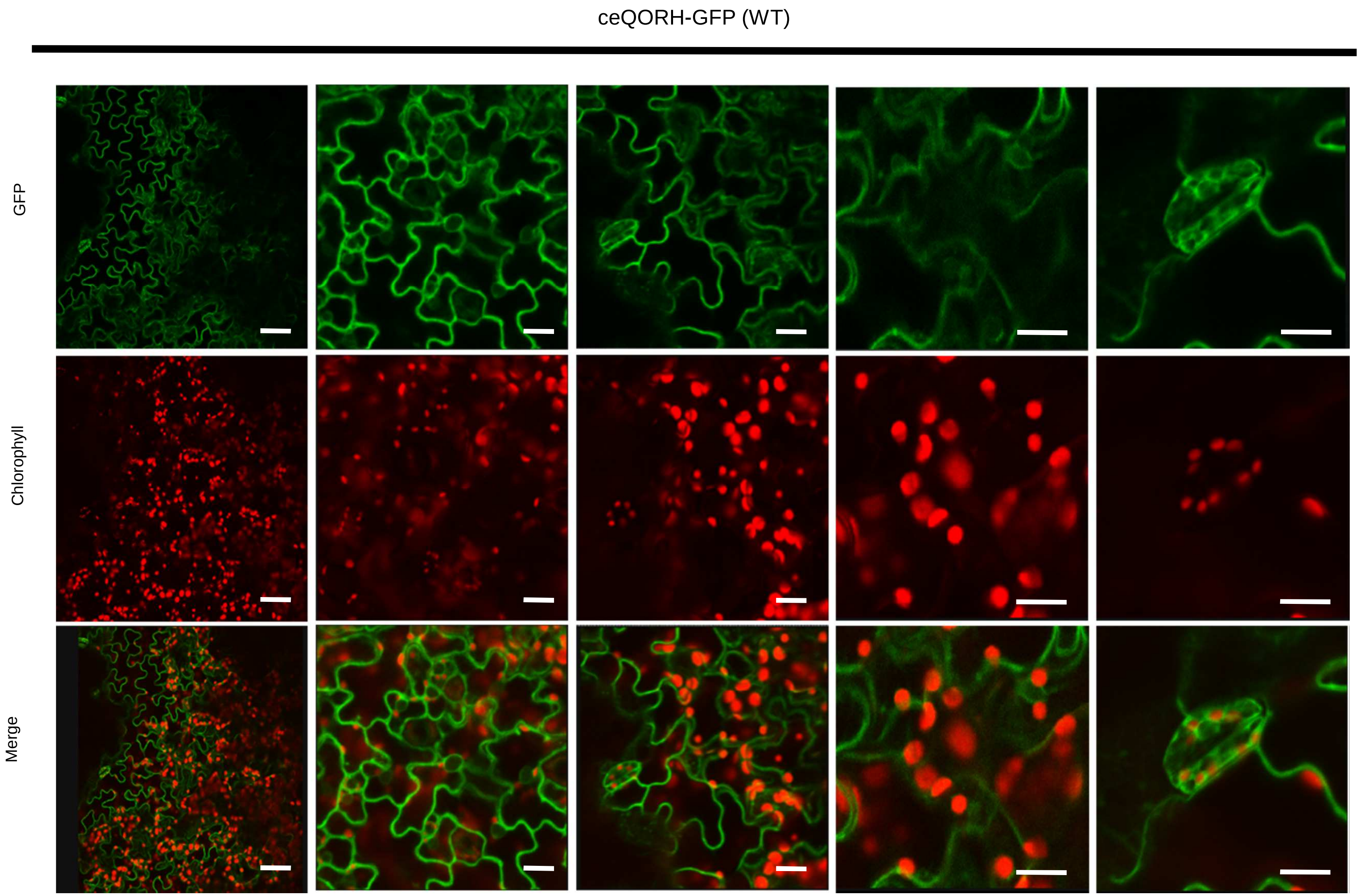

Supplementary Figure 3. ceQORH is not targeted to the chloroplast in epidermal cells from WT plants. Confocal microscopy was performed on WT plants stably expressing ceQORH-GFP (plant ceQORH fused to GFP). ceQORH-GFP; GFP fluorescence, Chlorophyll; chlorophyll autofluorescence. Merge: overlay of the two channels. Bar equals 40 µm in the first column, 20 in the second column and 10 µm in the three others.

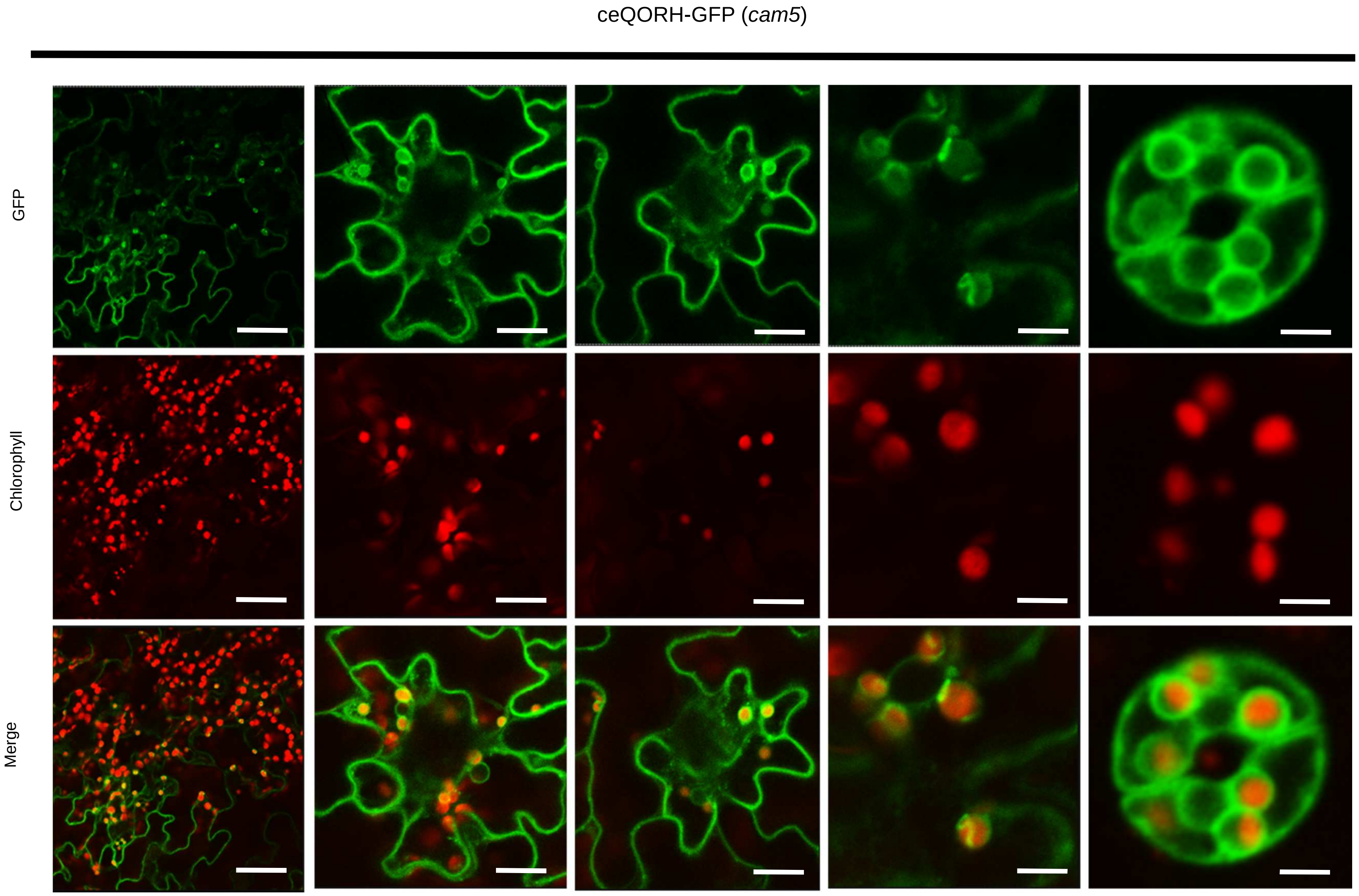

Supplementary Figure 4. ceQORH is targeted to the chloroplast in epidermal cells of the *cam5* mutant. Confocal microscopy was performed on both *cam5* mutant stably expressing ceQORH-GFP (plant ceQORH fused to GFP). ceQORH-GFP; GFP fluorescence, Chlorophyll; chlorophyll autofluorescence. Merge: overlay of the two channels. Bar equals 40 µm in the first column, 20 in the second and third columns and 5 µm in the two others.

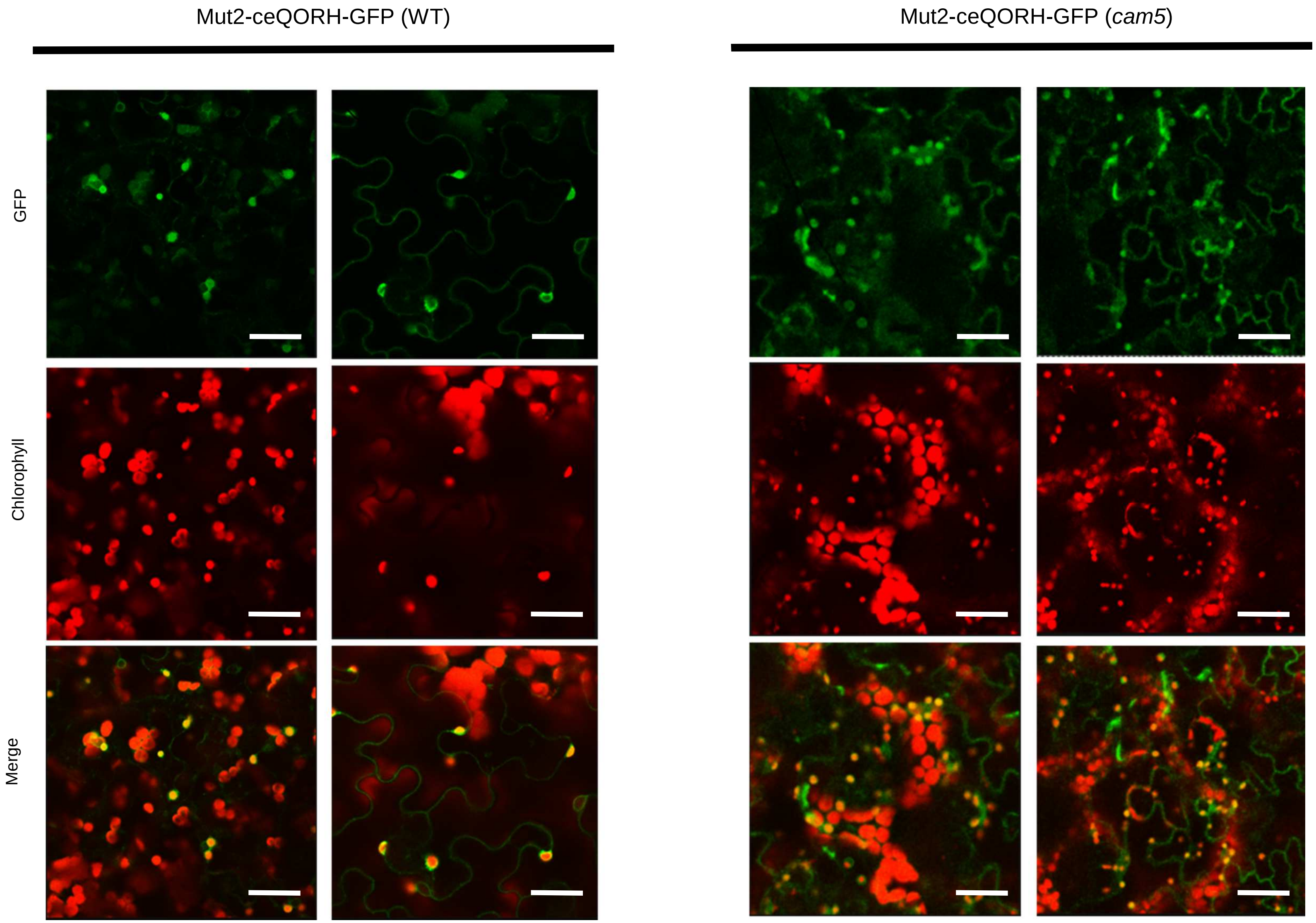

Supplementary Figure 5. ceQORH lacking CaM binding properties (Mut2-ceQORH) is targeted to the chloroplast in epidermal cells from both WT plants and the *cam5* mutant. Confocal microscopy was performed on both WT plants and cam5 mutant stably expressing Mut2-ceQORH-GFP (plant Mut2-ceQORH fused to GFP). Mut2-ceQORH-GFP; GFP fluorescence, Chlorophyll; chlorophyll autofluorescence. Merge: overlay of the two channels. Bar equals 20 µm.
